# Risk assessment of diesel exhaust and lung cancer: combining human and animal studies after adjustment for biases in epidemiological studies

**DOI:** 10.1186/1476-069X-10-30

**Published:** 2011-04-11

**Authors:** Xanthi Pedeli, Gerard Hoek, Klea Katsouyanni

**Affiliations:** 1 Department of Hygiene, Epidemiology and Medical Statistics, University of Athens, Medical School, Athens, Greece; 2 Institute for Risk Assessment Sciences, University of Utrecht, Utrecht, The Netherlands

## Abstract

**Background:**

Risk assessment requires dose-response data for the evaluation of the relationship between exposure to an environmental stressor and the probability of developing an adverse health effect. Information from human studies is usually limited and additional results from animal studies are often needed for the assessment of risks in humans. Combination of risk estimates requires an assessment and correction of the important biases in the two types of studies. In this paper we aim to illustrate a quantitative approach to combining data from human and animal studies after adjusting for bias in human studies. For our purpose we use the example of the association between exposure to diesel exhaust and occurrence of lung cancer.

**Methods:**

Firstly, we identify and adjust for the main sources of systematic error in selected human studies of the association between occupational exposure to diesel exhaust and occurrence of lung cancer. Evidence from selected animal studies is also accounted for by extrapolating to average ambient, occupational exposure concentrations of diesel exhaust. In a second stage, the bias adjusted effect estimates are combined in a common effect measure through meta-analysis.

**Results:**

The random-effects pooled estimate (RR) for exposure to diesel exhaust vs. non-exposure was found 1.37 (95% C.I.: 1.08-1.65) in animal studies and 1.59 (95% C.I.: 1.09-2.10) in human studies, whilst the overall was found equal to 1.49 (95% C.I.: 1.21-1.78) with a greater contribution from human studies. Without bias adjustment in human studies, the pooled effect estimate was 1.59 (95% C.I.: 1.28-1.89).

**Conclusions:**

Adjustment for the main sources of uncertainty produced lower risk estimates showing that ignoring bias leads to risk estimates potentially biased upwards.

## Background

Risk assessment is of principal importance in the determination of appropriate intervention measures to eliminate or prevent adverse health effects of environmental stressors in humans. While the literature on qualitative risk assessment is quite extensive, quantitative risk assessment of specific environmental stressors is more limited. Most risk assessments are grounded in the framework put forward by the National Research Council (NRC) in 1983 [1]. The framework distinguishes hazard identification, dose-response assessment, exposure assessment and risk characterization.

Quantitative risk assessment is hampered by a range of uncertainties, including limited data on dose-response functions [1,2]. Recently, the NRC updated the risk assessment framework and methodology focusing on the US Environmental Protection Agency practice [3]. Recommendations included better links between the risk management question and risk assessment design, more explicit account of uncertainty and variability and a harmonized approach for dose-response assessment [3].

One issue identified in the development of dose-response relationships for human health effects is the lack of human studies for many relevant exposures [3]. Ethical and practical problems often preclude this possibility, especially for rare diseases with long latency periods such as cancer. Furthermore, human studies are sometimes compromised by various biases. It is therefore desirable to take into account evidence from both human and animal studies.

A methodology for quantitative combination of human and animal studies has been proposed more than 20 years ago [4]. Using a Bayesian framework, the authors proposed to make use of dose-response slopes and the uncertainty derived from human and animal studies including different exposures (e.g. diesel engine emissions, coke oven emissions) and endpoints (e.g. lung cancer, mutagenesis). One conclusion from the study was that the use of animal data is more convincing when based upon studies from multiple similar substances and multiple species [4]. Their methodology has been applied in the assessment of the cancer risk of ionizing radiation in which human and animal studies on radon, uranium and other substances have been used to develop dose-response functions [5]. A more recent example of using a Bayesian framework involves the combination of animal and human data from chlorination byproducts [6].

When data from human and animal studies are quantitatively combined, biases in both types of studies need to be adjusted. Issues arise concerning the validity of available data (sources of systematic error in epidemiological and/or occupational studies) as well as the extrapolation from animal to human. Lack of data on confounding variables, selection bias and information bias are the main sources of bias in human studies. On the other hand, the extent to which rodent data may be useful for predicting human lung cancer risk of inhaled poorly soluble particles comprises a debated topic in the scientific community [7].

In the present study we illustrate a quantitative approach combining data from human and animal studies, adjusting for bias in human studies. We use the example of the assessment of lung cancer risk due to occupational exposure to diesel exhaust particles. The assessment of exposure to diesel emissions is difficult since they are highly complex mixtures and constitute only a portion of a broader mix of air pollutants. Almost the entire diesel particle mass (approximately 94%) is in the fine particle range of 2.5 microns or less in diameter [8]. Because of their small size, these particles can be inhaled and a portion will eventually become trapped within the small airways and the alveolar regions of the lung.

A persistent association of risk for lung cancer associated with diesel exhaust (DE) exposure has been observed in more than 30 epidemiologic studies published in the literature over the past 40 years. The majority of the epidemiologic studies evaluate distinct occupational groups. The remaining studies include re-analyses of specific studies and meta-analyses [9].

The body of epidemiologic evidence supports a causal association between exposure to DE and occurrence of lung cancer. The strength of association has been found weak to modest (RRs/ORs between 1.2 and 2.6 for exposed vs. unexposed), with a dose-response relationship observed in several studies. However, with the strongest risk factor for lung cancer being smoking, there is a lingering uncertainty as to whether smoking effects may be influencing the magnitude of the observed RRs. In studies in which the effects of smoking were controlled, increased RRs for the effect of DE on lung cancer prevailed.

Selection bias is certainly present in some of the occupational cohort studies that use external population data in estimating RRs, but this form of selection bias (a healthy worker effect) would only obscure, rather than spuriously produce, an association between DE and lung cancer. In effect, the usual standard mortality ratios observed in cohort mortality studies are likely to be underestimations of true risk [9].

Moreover, several other methodological limitations of individual studies have to be considered, such as small sample size, short follow-up period, lack of data on confounding variables, use of death certificates to identify the lung cancer cases, and lack of latency analysis. The studies with small sample sizes and short follow-up periods are hard to interpret due to these limitations. Some other uncertainties are methodological bias specifically characteristic of either cohort or case-control design, non-differential misclassification of exposure and/or outcome bias (i.e. use of inaccurate surrogates for diesel exposure and lung cancer incidence can lead to substantial bias) and exposure information bias which is certainly a problem for almost all of the studies considered [10].

The carcinogenic activity of diesel emissions has also been convincingly demonstrated in rats. More specifically, nearly lifetime exposure for at least 35 hours per week to high concentrations of DE particulate matter (2,000 - 10,000 μg/m^3^) causes an exposure-dependent increase in the incidence of benign and malignant lung tumors in rats. On the other hand, no consistent evidence suggests that diesel emissions induce lung cancer in mice and hamsters, which implies that species-specific factors play a critical role in the induction of lung tumors by diesel emissions [11].

Extrapolation of these results to humans involves several methodological limitations and caution is needed in the interpretation of findings. Specifically, the lung tumors observed in rats exposed to high concentrations of diesel emissions may be due to a species-specific response to inhaled particulate matter rather than to a carcinogenic mechanism that also occurs in humans. Moreover, extrapolation of no-effect levels for exposure to DE from one species to another is problematic because of wide intra-species variations in particle clearance rates and in susceptibility to cancer. Finally, the rat bioassay data do not exclude the possibility that DE may induce lung cancer by different mechanisms in different species, or by different mechanisms in the same species at different exposure levels [12,13].

The aim of the present study is to quantitatively combine evidence from selected human and animal studies of the association between exposure to DE and occurrence of lung cancer, after adjusting the reported risk estimates for the main source of bias in each study separately. This work is done under the framework of the INTARESE Project, a 5-year (2006-2010) integrated project, designed to support implementation of the European Environment and Health Action Plan [14].

## Methods

### Methods to Account for Bias in Human Studies

While random error decreases with increasing sample size, uncertainty about sources of systematic error remains. We focus here on characterizing quantitative aspects of uncertainty. More qualitative methods have been described before [15]. Quantitative methods, such as sensitivity analysis, Monte Carlo risk analysis (MCRA) and Bayesian uncertainty assessment are useful tools for a valuable insight into the importance of various sources of bias [16]. We focus here on bias due to confounding and misclassification of exposure.

We analyzed three human studies of the association between occupational exposure to DE and occurrence of lung cancer, to quantify the uncertainty potentially attributed to systematic error. Similarities between the types of exposure and between the outcomes under study were the basic criteria for the selection of the studies to be analyzed. We mainly focused on occupational studies since they comprise the bulk of the diesel epidemiology literature. Electronic searches were conducted with MEDLINE in order to identify studies published after 1990. Twenty-six studies were identified as potentially relevant.

Candidate studies should satisfy the following criteria: (1) estimates of relative risks (including standardized mortality ratios and odds ratios) and their standard errors should be available; (2) studies should have an adequate latency period (at least 10 years) for the development of lung cancer after the exposure's onset; (3) studies should cover similar or overlying time periods; (4) studies should be conducted in different populations/areas; (5) studies should not suffer from important uncertainties that could render them unreliable, but some bias should be obvious. Some of these criteria have also been used in the past by Lipsett and Campleman [17].

The three studies finally selected [18-20] satisfy all of the above criteria. We have avoided a systematic review. This is because the main interest lies rather on the demonstration of the methods suggested than on the derivation of a combined estimate based on all available evidence. Though we do not pretend that the three selected epidemiological studies are representative for the entire body of epidemiological studies, the RRs agree well with the indicative relative risk of 1.4 comparing diesel-exposed versus non-exposed used in an indicative EPA assessment [21]. A recent large study that pooled a large number of case control studies reported an odds ratio of 1.31 comparing the highest exposed versus non-exposed subjects [22].

For each study we identified the most important source of bias that may affect the prominent outcome (i.e. lung-cancer disease onset or mortality) and quantified such sources by applying ordinary sensitivity analysis [23] or a simulation-based process [24]. In most cases, the study's design indicates the potential sources of systematic error. Furthermore, the authors themselves usually recognize and mention the drawbacks of their study and frequently they prioritize them too. However, there are also cases where it is not clear at all which one of the available sources of bias is the most significant or there are more than one sources with the same rank of significance. In such cases, the characterization of a particular source of bias as the most important is rather subjective. The studies selected for the current analysis are well-designed and most of their drawbacks are usually inevitable in the framework of occupational cohort studies. The motivations for deciding the most important sources of systematic error are described in detail in section 4.1.

Ordinary sensitivity analysis was used for the quantification of potential confounding effects while a simulation-based process was employed for quantifying misclassification bias. Ordinary or traditional sensitivity analysis estimates what the true effect measure (e.g. rate ratio) would be in light of the observed data and some hypothetical level of bias and it produces one or more adjusted point estimates for the effect measure of interest. The general strategy begins with the addition of omitted sources of uncertainty as free parameters or their use to adjust the data. Conventional analysis is then repeated with these parameters set at fixed values, as if they were known. The resulting array of effect estimates is examined for consistency or for patterns of dependency on the varied parameters [23].

An effective approach to account for uncertainty in the exposure assignment is the application of an iterative simulation process. The process begins with the specification of the simulation parameter values. The classification probabilities are assumed to be the same for cases and non-cases so that the misclassification process is non-differential. The number of resultant combinations determines the number of repetitions. For a given set of simulation experiment parameters, a dataset is generated based on the usual assumption that the random error follows a binomial distribution. On each simulation trial, we use the ratios of the randomly generated numbers of cases to the fixed number of individuals in each exposure group to calculate what the true effect estimate for that trial would have been were there no misclassification. The numbers of false-negative and false-positive individuals are calculated using binomial random variates. Finally an effect estimate with individuals misclassified on exposure status is calculated at each iteration using the misclassified counts [24].

### Extrapolation from Animal to Human

Historically, to estimate low-dose effects, regulators have used the so-called no observed effect levels (NOELs), no observed adverse effect levels (NOAELs), lowest observed adverse effect levels (LOAELs) etc. An important drawback of observed effect level (OEL) estimation is that it is tied critically to the spacing of doses chosen for each study. The Benchmark dose (BMD) is an alternative to OELs that is not restricted to the experimental levels [25].

The BMD approach involves modeling the dose-response curve in the range of the observed data, and then using that model to interpolate an estimate of the dose that corresponds to a particular level of response. A measure of uncertainty is also calculated, e.g. a confidence limit or Bayesian posterior. The lower confidence limit of the BMD, called BMDL, accounts for the uncertainty in the estimate of the dose response that is due to characteristics of the experimental design (e.g. sample size). The BMDL is used as the basis for the point of departure for linear low-dose extrapolation, the dose-response assessment approach applied to most carcinogens.

We adopted the BMD approach to improve the extrapolation procedure, with an appropriate point of departure for linear extrapolation at low doses. The BMD approach does not remove the need for assumptions about the appropriateness of extrapolation from animals to humans. The most serious and still questionable assumption is that of the relevance of species-specific responses to inhaled particles for the estimation of human lung cancer risk.

Three DE-related rat studies on lung tumorigenicity comprise the group of animal studies to be analyzed. In fact, we decided to focus on rats since previous studies have shown that they are more appropriate for assays of the inhalation carcinogenicity hazard of particles and mixtures containing particles [26]. For the purposes of our analysis, a benchmark response (BMR) of 10% extra risk was used, since it is typical for standard cancer bioassay data [25]. Selection of models' degrees was based on AIC and on likelihood ratio tests for the evaluation of the improvement in fit afforded by estimating additional parameters.

Having calculated a BMD for each study, its 95% lower confidence limit (BMDL) was used as the point of departure for linear extrapolation at low doses corresponding to average ambient and occupational exposure concentrations of the pollutant under study. So, in each dataset we fitted a simple linear regression model of the form *y = β*_*0 *_*+ β*_*1*_*x *where *x *and *y *represent the DE concentration (μg/m^3^) and the corresponding fraction of rats affected. Thus, the BMD analysis' results are used as input for this model. More specifically, for the estimation of the intercept *β*_*0 *_and the slope *β*_*1 *_we use information about two pairs (*x, y*): (i) the fraction of non-exposed rats affected (i.e. *x *= 0, *y *as reported in the original study) and (ii) the fraction affected at a concentration equal to the BMDL (i.e. *x *= BMDL, *y *pre-specified at 0.10 by design). Risk ratio estimates were finally derived using the formula , where *d *represents the average ambient concentration of the pollutant under study.

## Data

### Human data

Table 1 summarizes the three selected human studies. All of these studies concern occupational cohorts and the estimated lung cancer risk ratios range between 1.40 and 2.18 comparing diesel exposed and non-exposed subjects. Confounding due to smoking has been identified as the major source of bias in the studies [18] and [19], while misclassification bias is the most important source of systematic error in the study [20].

**Table 1:** Selected Diesel Exhaust - Related Occupational Studies on Lung Cancer Incidence/Mortality.

Study	Effect Measure	**95% C.I**.	Main Source of Systematic Error
Garshick et al. [18]*(retrospective cohort)*	RR = 1.40^a^	1.30 - 1.51	Confounding by smoking
Wellmann et al. [19]*(retrospective cohort)*	SMR = 2.18 ^a^	1.61 - 2.87	Confounding by smoking
Gustavsson et al. (20] *(case-referant)*	RR = 1.63^b^	1.14 - 2.33	Misclassification bias

^a ^comparing occupationally exposed versus non-exposed

^b ^comparing the highest diesel related NO2 category versus unexposed

Garshick et al. [18] conducted a large cohort study aiming to assess lung cancer mortality in U.S. railroad workers between 1959 and 1996. For their purpose, they used data for 4,973 white males aged 40-64 years in 1981 with 10-20 years of railroad service in 1959. The level of occupational exposure to DE was determined by the job type (engineers, conductors, shop-workers and unexposed). Exposure duration was calculated in years from 1959 to retirement and expressed in terms of a categorical variable with six levels: 0 (unexposed, i.e. workers who remained unexposed during the entire period of follow-up), 1-<5, 5-<10, 10-<15, 15-<20, 20 years. Lung cancer was identified as the underlying cause in 4,021 of deaths and as a contributing cause in 330 of deaths. Disregarding exposure in the five years before death, the RR for diesel exposed workers compared with workers without regular work in an exposed job was 1.40 (95% C.I.: 1.30-1.51). The data did not provide evidence of an increased risk with increasing years of work in a job with exposure to DE.

In this cohort, individual data on smoking history were not available. Thus, the authors followed indirect approaches in order to minimize the possible effect of uncontrolled confounding by smoking. Firstly, they tried to include only workers of similar socioeconomic status since its correlation with smoking habits is well-known in the literature (see e.g. [27]). Secondly, the observed lung cancer relative risks were divided by age- and job-specific smoking adjustment factors [28]. As the authors note, this indirect method is limited in adjusting the smoking by assuming no interaction between diesel exposure and smoking. However, the available data were insufficient to assess this possibility. Thus, it is probable that confounding by smoking remains partly uncontrolled.

Wellmann et al. [19] used data on 2,053 blue-collar workers of a large carbon black plant in Germany to investigate the association between occupational exposure to carbon black and mortality. Workers should be continuously employed for at least one year between 1 January 1960 and 31 December 1998. Those who were hired before 1960 but fulfilled the inclusion criteria were also eligible (census cohort). Exposure levels were defined by assigning scores of exposure to each job title. The highest score (20 units) was assigned to jobs where carbon black had to be shoveled into bags. A score of zero was assigned to the few jobs with no contact with carbon black. The study's results support a more than twofold increase of lung cancer mortality in the census cohort (SMR = 2.18, 95% C.I.: 1.61-2.87).

Compared with other epidemiological studies on occupational hazards, information on smoking was relatively complete in this study. More specifically, information on smoking was obtained by occupational physicians for 77% of the members of the cohort. However, as noted by the authors, this source of data may have introduced an under-reporting bias because workers may have had reservations about report smoking habits correctly. Moreover, smoking data are essentially limited to the subjects' period of employment in the plant under study. Thus, categorization of smoking intensity may also be subject to misclassification due to the limited time period for which information is available. Taking into account all these limitations we assume that the estimated SMRs may be partly affected by the inadequate control for the confounding effect of smoking. This rationale motivates a further analysis of the impact of more detailed confounding control.

The main objective of Gustavsson et al. study [20] was to investigate the lung cancer risk from occupational exposure to DE, mixed motor exhaust, other combustion products, asbestos, metals, oil mist and welding fumes. As cases were used all men aged 40-75 years who were residents of Stockholm County, Sweden, at any time between 1985 and 1990 and who had lived outside the county for no more than 5 years during the period 1950-1990. Referents were selected at random from the general population and were frequency-matched to the cases with regard to age (in 5-year groups) and year of inclusion (1985-1990). Response rates of 87% and 85% resulted in 1,042 cases and 2,364 referents, respectively. Nitrogen dioxide was used as an indicator for exposure to DE (alone or in combination with gasoline exhaust). For each pollutant, four exposure levels were defined. For DE (μg of NO_2_/m^3^) exposure levels were the following: unexposed, 40-119, 120-399, > = 400. Risk estimates were adjusted for tobacco smoking, other occupational exposures, residential radon and environmental exposure to traffic-related air pollution. For the highest quartile of cumulative exposure versus no exposure, the relative risk was 1.63 (95% C.I.: 1.14-2.33). As mentioned by the authors, error in the exposure assessment, such as systematic over- or under-estimation of historical exposure levels, is probably the most significant methodological problem of the study. Detailed smoking data were available.

### Animal data

Four basic criteria were used for the selection of the particular animal studies. Firstly, we considered the type of exposure and focused on studies that were examining rodents exposed to DE soot-associated organic compounds. Secondly, we were interested in the induction of lung tumors so as to keep a kind of consistency with human studies where the occurrence of lung cancer was studied. The third criterion regards the species under consideration. We focused on rat studies since, as already noted, consistent evidence suggests that diesel emissions induce lung tumors in rats but such evidence for mice and hamsters is rather ambiguous. Finally, we needed studies where information about the number of animals examined and the number of animals with lung tumors was available.

Thus, from the various outcomes assessed in each one of the selected animal studies we focused on lung carcinogenicity. The subjects of all studies were rats, except from the study [29] where both rats and Syrian hamsters were participating. In this case, only results regarding tumors in the lungs of rats were re-analyzed. The different DE exposure concentrations were standardized into a common metric which is proportional to cumulative exposure. The resulting metric is the 30-month average continuous exposure expressed in units of micrograms per cubic meter. For example, 0.7 mg/m^3^, 80 hours/week, for 24 months equals [(700 *μg*/*m*^3^) (80 *hours*/*week*) (24)] [(168 *hours*/*week*) (30)] = 270 *μg*/*m*^3^, continuous 30-month exposure [30]. Table 2 summarizes the three studies under consideration in terms of pollutant's concentrations, number of animals examined and number of animals with lung tumors.

**Table 2:** Selected DE^a^-Related Rat Studies on Lung Tumorgenicity.

Study	Exposure Duration	**DE concentration during exposure (mg/m**^**3**^**)**	**30-month average continuous concentration (μg/m**^**3**^**)**	No. of animals examined	No. of animals with lung tumors	% of animals with lung tumors
Nikula et al. [31]	80 hours/week,	0	0	214	3	1.4
	24 months	2.44	930	210	17	8.1
		6.33	2400	212	55	25.9

Brightwell et al. [29]	80 hours/week	0	0	250	4	1.6
	24 months	0.70	270	112	1	8.9
		2.20	840	112	14	12.5
		6.60	2500	111	55	49.6

Mauderly et al. [32]	35 hours/week	0	0	230	2	0
	30 months	0.35	73	223	3	0.35
		3.5	730	222	8	3.5
		7.08	1,480	227	29	7.08

^a ^DE: diesel exhaust

Nikula et al. [31] investigated the importance of the DE soot - associated organic compounds in the lung tumor response of rats. Male and female, five to seven week old F344 rats were exposed chronically to diluted whole DE or aerosolized carbon black (CB) 16 hours/day, five days/week at target particle concentrations of 2.5 mg/m^3 ^(LDE, LCB) or 6.5 mg/m^3 ^(HDE, HCB) or to filter air. The CB served as a surrogate for the elemental carbon matrix of DE soot. The rats were assigned randomly to the five treatment groups by randomizing each gender by body weight measured nine days before the start of exposures. The rats were seven to nine weeks old when the exposure began. Approximately, 100 rats of each gender per treatment group were observed for life span to evaluate body weight, survival and carcinogenicity. The exposures were terminated at 24 months. According to the study findings, both DE and CB particles accumulated progressively in the lungs of exposed rats, but the rate of accumulation was higher for DE soot. In general, DE and CB caused similar, dose-related, non-neoplastic lesions. Logistic regression modeling did not demonstrate significant differences between the carcinogenic potencies of CB and DE in either gender. The results suggested that the organic fraction of DE may not play an important role in the carcinogenicity of DE in rats.

Brightwell et al. [29] conducted a chronic inhalation study in order to examine the potential carcinogenic effect of inhaled automobile exhaust emissions in rodents. The animals used were Fischer 344 rats and Syrian golden hamsters (Charles River, MA, USA) and were six to eight weeks old at the start of exposure. Both rats and hamsters were exposed to the emissions from i) a gasoline engine, ii) a gasoline engine fitted with a three-way catalytic converter, iii) a diesel engine and iv) a diesel engine with particle filtration. Exposures lasted for two years and were for 16 hours per day, five days per week. The animals were grouped in ten experimental groups. Three concentration levels were used for the diesel-exposed animals. The mean particle concentration in the diesel engine chambers was 0.7, 2.2 and 6.6 mg/m^3 ^for the low-, medium- and high- concentration chambers, respectively. A further group of control animals were exposed to conditioned air only. Each experimental group consisted of 144 rats and 624 hamsters. The control group was comprised by 288 rats and 624 hamsters. Each group contained an equal number of male and female animals. Interim sacrifices of rats and non-pretreated hamsters were carried out 6, 12, 16, 18 and 24 months. Exposure effects were measured by means of changes in body and organ weights and incidence of tumours of the respiratory tract. According to the findings, there was a significant increase in the incidence of lung tumours in Fischer 344 rats exposed to diesel engine emissions compared with unexposed controls. The increased tumour incidence was only evident in the medium- and high-dose level exposure groups, but there was a clear indication of a dose response at these two levels. No evidence for any increase in lung tumours in rats or in hamsters exposed to gasoline, gasoline catalyst or filtered diesel exhaust arose.

The aim of Mauderly et al. [32] was to investigate whether chronic inhalation of diesel exhaust is a pulmonary carcinogen in rats. For this purpose, male and female specific pathogen-free F344/Crl rats were randomized by litter into four treatment groups. More specifically, the rats were exposed seven hours/day, five days/week for up to 30 months to whole exhaust diluted to nominal soot concentrations of 0.35 (low), 3.5 (medium), or 7.0 (high) mg/m^3 ^or to filtered air as sham-exposed controls. Rats surviving to 30 months of exposure were terminated for histopathology. The remaining either died spontaneously or were euthanized when found moribund. Of a total of 364-367 rats entered into each treatment group, 221-230 rats dying, euthanized, or terminated were examined for lung tumours. Body weight and survival were not affected by exposure. A progressive accumulation of soot in the lung was accompanied by a focal fibrotic and proliferative lung disease. The four tumour types observed (adenoma, adenocarcinoma, squamous cyst and squamous cell carcinoma) were all of epithelial origin and their prevalence was significantly higher at the medium (4%) and high (13%) dose levels as compared to the control group (1%). Finally, a significant relationship between tumour prevalence and both exposure concentration and soot lung burden was indicated. According to the study findings, the authors concluded that chronic inhalation of diesel exhaust at a high concentration is a pulmonary carcinogen in the rat.

## Results

### Adjustment for bias in human studies

To account for the potential confounding effect of smoking in the corresponding occupational studies of the effects of DE on lung cancer mortality, we applied sensitivity analyses under alternative scenarios for the smoking prevalence in the occupational cohorts and the respective general populations (reference groups) and for the lung cancer rate ratios of smokers vs. non-smokers. Estimates of these measures were obtained from related studies or published data with respect to the study periods and populations. Smoking adjusted SMRs comparing DE-exposed versus non-exposed were calculated as the ratios of observed SMRs and appropriate bias factors.

For the study [18], smoking prevalence was assumed to be 47.1% in the occupational cohort and 40.9% in the general population while the lung cancer rate ratio of smokers vs. non-smokers was considered to range between 14 and 19. The 1985 Report of the Surgeon General [33] was the source of the above estimates. More specifically, we used the percentages for 1978-1980 because this period coincides with the middle of our 38-years study period. According to the results summarized in Table 3, the observed lung cancer SMR of 1.40 would reduce to 1.24 under the evaluated scenarios, indicating a substantial bias. There was little difference between bias factors between the various scenarios.

**Table 3:** Ordinary Sensitivity Analysis for the Garshick et al. study [18].

**RR**_**smoking**_^**c**^	**I**_**nonexp**_^**d**^	**I**_**exp**_^**e**^	**bias factor**^**f**^	**RR**_**adj**_^**g**^	**95% C.I**.
14	6.317I_0_^h^	7.123I_0_	1.128	1.242	1.153-1.339
15	6.726I_0_	7.594I_0_	1.129	1.240	1.151-1.337
16	7.135I_0_	8.065I_0_	1.130	1.239	1.150-1.336
17	7.544I_0_	8.536I_0_	1.131	1.237	1.149-1.335
18	7.953I_0_	9.007I_0_	1.133	1.236	1.148-1.333
19	8.362I_0_	9.478I_0_	1.133	1.235	1.147-1.332

Adjusted lung cancer rate ratios for several scenarios about the RR for ever smokers vs.

no-smokers, assuming RR_obs_^a ^= 1.40 (95% C.I.: 1.30 - 1.51) for DE^b ^exposure and smoking prevalence equal to 47.1% and 40.9% in the occupational cohort and the general population respectively.

^a ^RR_obs_: observed risk ratio

^b ^DE: diesel exhaust

^c ^RR_smoking_: risk ratio of smokers vs. non-smokers

^d ^I_nonexp_: lung cancer mortality rate of non-DE exposed workers due to smoking

^e ^I_exp_: lung cancer mortality rate of DE exposed workers due to smoking

^f ^bias factor = I_exp_/I_nonexp_

^g ^RR_adj_: adjusted risk ratio

^h ^I_0_: lung cancer mortality rate of non-smokers

Regarding the study [19], we assigned a smoking prevalence of 84% and of 65%-77% (using a 2% step) to the occupational cohort and reference population respectively. We also assumed a lung cancer rate ratio of smokers vs. non-smokers equal to 10.7 or 12 in turn. The appropriate information was derived from two studies published afterwards in the study population [34,35]. Adjusted rate ratios under the resultant 14 different scenarios are summarized in Table 4. The analysis showed that the lung cancer SMR of 2.17 reduced to 1.74 - 2.02 after control for confounding in the various scenarios.

**Table 4:** Ordinary Sensitivity Analysis for the Wellmann et al. study [19].

**RR**_**smok**_	***Pr***^***(r)***^	**I**_**nonexp**_	**I**_**exp**_	bias factor	**SMR**_**adj**_	**95% C.I**.
10.7	0.65	7.31I_0_	9.15I_0_	1.252	1.741	1.286-2.292
12.0	0.65	8.15I_0_	10.24I_0_	1.256	1.735	1.281-2.284
10.7	0.67	7.50I_0_	9.15I_0_	1.220	1.787	1.320-2.353
12.0	0.67	8.37I_0_	10.24I_0_	1.223	1.782	1.316-2.346
10.7	0.69	7.69I_0_	9.15I_0_	1.189	1.833	1.354-2.414
12.0	0.69	8.59I_0_	10.24I_0_	1.192	1.829	1.351-2.408
10.7	0.71	7.89I_0_	9.15I_0_	1.160	1.879	1.388-2.474
12.0	0.71	8.81I_0_	10.24I_0_	1.162	1.876	1.385-2.469
10.7	0.73	8.08I_0_	9.15I_0_	1.132	1.926	1.422-2.535
12.0	0.73	9.03I_0_	10.24I_0_	1.134	1.922	1.420-2.531
10.7	0.75	8.28I_0_	9.15I_0_	1.105	1.972	1.456-2.596
12.0	0.75	9.25I_0_	10.24I_0_	1.107	1.969	1.454-2.593
10.7	0.77	8.47I_0_	9.15I_0_	1.080	2.018	1.490-2.657
12.0	0.77	9.47I_0_	10.24I_0_	1.081	2.016	1.489-2.654

Adjusted rate ratios for several scenarios about the smoking prevalence in the general population (*Pr*^*(r)*^) and assuming a lung cancer RR for ever smokers vs. non-smokers equal to 10.7 or equal to 12 (SMR_obs_^a ^= 2.18 (95% C.I.: 1.61 - 2.87), smoking prevalence in the occupational cohort 84%).

^a ^SMR: standardized lung cancer mortality rate comparing (diesel exposed) workers versus the general population (see table 3 for further definitions).

An iterative simulation process was applied to the study [20] where 373 referents were exposed to DE and the probability of exposure for each work period and substance was assessed as 0, 20, 50, or 85 percent, i.e. no cases (0%) or 208 cases (20%) or 521 cases (50%) or 886 cases (85%). Based on this information, we considered four different alternatives for the exposure prevalence: (i) *P*_*E *_= 0.10(≈ 373/3,406), (ii) *P*_*E *_= 0.16 (≈(373 + 208)/3,406), (iii) *P*_*E *_= 0.25 (≈(373 + 521)/3,406), (iv) *P*_*E *_= 0.36 (≈(373 + 886)/3,406).

The unexposed group consisted of 842 cases and 1,991 controls. Moreover, the study population comprised all men aged 40-75 years who were residents of Stockholm County, Sweden, at any time between 1985 and 1990 and exposure assessment went back to 1950. So the study period for each individual varies between 35 and 40 years. Taking the average (37.5 years), we estimated the incidence proportion in the unexposed individuals as *I*_0 _= 842/[37.5* (842 + 1,991)]≈ 0.8%. Sensitivity and specificity were set alternatively equal to 0.4, 0.6, 0.8 and 1 (nine combinations).

Observed relative risk was not summarized in a single estimate due to the existence of more than one exposure groups. More specifically, the researchers estimated a RR equal to 0.65 (95% C.I.: 0.40 - 1.04) for the first exposure group (>0-0.53 μg-years/m^3 ^NO_2_), 1.13 (95% C.I.: 0.77 - 1.66) for the second (0.54-1.41 μg-years/m^3 ^NO_2_), 1.05 (95%C.I.: 0.70 - 1.60) for the third (1.42-2.37 μg-years/m^3 ^NO_2_) and 1.63 (95% C.I.: 1.14 - 2.33) for the last exposure group (>2.38 μg-years/m^3 ^NO_2_). In our analysis we used all these estimates in turn. We present results for only the highest quartile of cumulative exposure (>2.38 μg-years/m^3 ^NO_2_). For this exposure group, initial results indicated a significant increase in the lung cancer risk due to occupational exposure to DE, while non-significant effects have been estimated for the other exposure groups. Results for all exposure groups are available upon request.

Combining all the different scenarios described above, lead to a total of 4 × 3 × 4 × 4 = 192 simulation experiments. In each experiment, 10,000 trials (iterations) were conducted in order to estimate a RR adjusted for misclassification bias. Median misclassified estimates by exposure prevalence, sensitivity and specificity are presented in Table 5. Depending on the degree of exposure misclassification, the bias adjusted risk ratios ranged between 1.70 and 1.98.

**Table 5:** Bias Analysis of the Gustavsson et al. study [20].

**P**_**E**_	Se	Sp	**Mean **^**b**^	**95% C.I**.
0.10	1.00	1.00	1.70	1.19 - 2.42
0.10	1.00	0.80	1.72	1.20 - 2.45
0.10	1.00	0.60	1.73	1.19 - 2.43
0.10	1.00	0.40	1.70	1.19 - 2.45
0.10	0.80	1.00	1.71	1.21 - 2.47
0.10	0.80	0.80	1.70	1.22 - 2.47
0.10	0.80	0.60	1.72	1.20 - 2.47
0.10	0.80	0.40	1.72	1.20 - 2.48
0.10	0.60	1.00	1.76	1.22 - 2.52
0.10	0.60	0.80	1.76	1.23 - 2.52
0.10	0.60	0.60	1.71	1.23 - 2.47
0.10	0.60	0.40	1.72	1.21 - 2.47
0.10	0.40	1.00	1.83	1.28 - 2.66
0.10	0.40	0.80	1.79	1.25 - 2.57
0.10	0.40	0.60	1.78	1.23 - 2.54
0.10	0.40	0.40	1.74	1.21 - 2.53
0.16	1.00	1.00	1.70	1.19 - 2.44
0.16	1.00	0.80	1.72	1.20 - 2.47
0.16	1.00	0.60	1.71	1.21 - 2.44
0.16	1.00	0.40	1.72	1.20 - 2.46
0.16	0.80	1.00	1.73	1.22 - 2.46
0.16	0.80	0.80	1.74	1.22 - 2.50
0.16	0.80	0.60	1.73	1.20 - 2.47
0.16	0.80	0.40	1.74	1.21 - 2.47
0.16	0.60	1.00	1.76	1.25 - 2.49
0.16	0.60	0.80	1.77	1.23 - 2.54
0.16	0.60	0.60	1.74	1.22 - 2.50
0.16	0.60	0.40	1.74	1.22 - 2.48
0.16	0.40	1.00	1.88	1.32 - 2.65
0.16	0.40	0.80	1.82	1.29 - 2.57
0.16	0.40	0.60	1.80	1.24 - 2.55
0.16	0.40	0.40	1.76	1.23 - 2.51
0.25	1.00	1.00	1.73	1.20 - 2.45
0.25	1.00	0.80	1.73	1.21 - 2.46
0.25	1.00	0.60	1.73	1.20 - 2.47
0.25	1.00	0.40	1.72	1.20 - 2.49
0.25	0.80	1.00	1.74	1.22 - 2.49
0.25	0.80	0.80	1.75	1.23 - 2.51
0.25	0.80	0.60	1.76	1.22 - 2.51
0.25	0.80	0.40	1.74	1.21 - 2.49
0.25	0.60	1.00	1.79	1.26 - 2.55
0.25	0.60	0.80	1.78	1.25 - 2.56
0.25	0.60	0.60	1.78	1.24 - 2.52
0.25	0.60	0.40	1.76	1.23 - 2.51
0.25	0.40	1.00	1.91	1.31 - 2.69
0.25	0.40	0.80	1.85	1.29 - 2.64
0.25	0.40	0.60	1.81	1.27 - 2.57
0.25	0.40	0.40	1.76	1.24 - 2.53
0.36	1.00	1.00	1.73	1.22 - 2.48
0.36	1.00	0.80	1.75	1.22 - 2.50
0.36	1.00	0.60	1.75	1.22 - 2.50
0.36	1.00	0.40	1.75	1.22 - 2.50
0.36	0.80	1.00	1.76	1.24 - 2.51
0.36	0.80	0.80	1.77	1.23 - 2.53
0.36	0.80	0.60	1.76	1.24 - 2.51
0.36	0.80	0.40	1.75	1.23 - 2.52
0.36	0.60	1.00	1.82	1.27 - 2.61
0.36	0.60	0.80	1.80	1.27 - 2.60
0.36	0.60	0.60	1.79	1.26 - 2.56
0.36	0.60	0.40	1.78	1.25 - 2.55
0.36	0.40	1.00	1.98	1.39 - 2.79
0.36	0.40	0.80	1.90	1.33 - 2.69
0.36	0.40	0.60	1.84	1.28 - 2.63
0.36	0.40	0.40	1.80	1.25 - 2.57

estimates by exposure prevalence (P_E_), sensitivity (S_E_) and specificity (S_p_) where *RR*_*T *_^a ^= 1.63 (95% C.I.: 1.14 - 2.33) and incidence proportion rate in unexposed subjects equals 0.008.

^a ^RR_T _: true relative risk

^b ^RR_M _: estimated relative risk having adjusted for misclassification

### Extrapolation from animal studies

In order to obtain human equivalent effect estimates, we used the BenchMark Dose Software (BMDS) Version 2.0 [25]. In each study, for the estimation of the benchmark dose (BMD) and its lower confidence limit (BMDL) we applied a multistage model with a BMR of 10% extra risk. The lower AIC's of the second-degree models together with smaller standardized residuals as compared to the corresponding first-degree models, lead to the choice of second-degree multistage models for all the DE-related animal studies.

Having calculated a BMD for each study, its BMDL was then used as the point of departure (POD) for linear extrapolation at low doses corresponding to average ambient concentrations of the pollutant under study. More specifically, the estimated BMDLs were extrapolated at the 65 μg/m^3^, the 1997 24-hour fine particle standard of the European Protection Agency. The resulting estimated effect estimates range from 1.25 to 1.58 (Table 6).

**Table 6:** Estimated *RR*s from rat exposure studies using BMDLs as the points of departure for linear extrapolation.

Study	**BMD**^**a **^**(μg/m**^**3**^**)**	**BMDL**^**b **^**(μg/m**^**3**^**)**	^**c**^	**95% C.I**.
Nikula et al. [31]	1289.730	936.256	1.426	0.95-1.90
Brightwell et al. [29]	963.542	716.572	1.246	0.83-1.67
Mauderly et al. [32]	1376.890	1183.880	1.576	0.84-2.31

^a ^BMD: benchmark dose (dose with a risk of 10%)

^b ^BMDL: 95% lower confidence limit of BMD

^c ^using the US 1997 24-hour fine particle standard of 65 μg/m^3^

### Combining evidence from human and animal studies

The bias-adjusted effect estimates from human and animal studies are summarized in Table 7. Meta-analysis of these estimates seems reasonable since they are quite consistent. Note that apart from justifying meta-analysis, consistency between human and animal bias-adjusted risk estimates indicates the effectiveness of the extrapolation from animal to human.

**Table 7:** Individual study and pooled bias-adjusted effect estimates comparing diesel exposed versus non-exposed and their 95% C.I.'s.

Study		Bias Adjusted Estimate	**95% C.I**.
*Human studies *			

Garshick et al. [18]		1.24	1.16 - 1.31
Wellmann et al. [19]		1.88	1.57 - 2.19
Gustavsson et al. [20]		1.76	1.15 - 2.37
**Pooled**	Fixed^a^	1.279	1.207 - 1.351
	Random	1.594	1.086 - 2.101
	Random without bias adjustment	1.727	1.218 - 2.236
*Animal studies *			

Nikula et al. [31]		1.43	0.95 - 1.90
Brightwell et al. [29]		1.25	0.83 - 1.67
Mauderly et al. [32]		1.58	0.84 - 2.31
P**ooled**	Fixed	1.365	1.075 - 1.654
	Random	1.365	1.075 - 1.654
*All studies (pooled estimate)*			

Fixed^a^		1.284	1.215 - 1.354
Random		1.492	1.210 - 1.775
Random without bias adjustment		1.586	1.279 - 1.892

^a ^significant heterogeneity

As a first step to the integration of all the available information, human and animal studies were separately meta-analyzed. Significant heterogeneity was found among the human studies with a random-effects pooled estimate equal to 1.73 (95% C.I.: 1.22 - 2.34) before bias adjustment. After bias adjustment, the pooled effect was 1.59 (95% C.I.: 1.09 - 2.10). In contrast, the meta-analysis of animal studies did not reveal significant heterogeneity and the pooled estimate was found equal to 1.37 (95% C.I.: 1.08 - 1.65).

Finally, the bias-adjusted effect estimates from human and animal studies were meta-analyzed in order to obtain a combined effect estimate. As expected, results gave evidence for significant heterogeneity (p-value = 0.001). The random-effects pooled estimate was found equal to 1.49 (95% C.I.: 1.21 - 1.78) with a greater contribution from human studies (Table 7). Without adjustment for bias in the various human studies, the random-effects pooled estimate was 1.59 (95% C.I.: 1.28 - 1.89), illustrating the importance of bias adjustment. It is also interesting to note that the effect estimates obtained from the pooled analysis of human studies only and from the meta-analysis without bias adjustment are both around 1.59. However, the beneficial effect of bias-adjustment is depicted in the 95% confidence intervals which are obviously narrower after the bias-adjustment.

## Discussion

The lack of adequate human exposure-response data renders the use of animal data essential in order to assess the harmful activity of specific environmental stressors in humans. Quantitatively combining evidence from both human and animal studies makes full use of all the relevant information. However, available techniques for such an approach are rather limited. The methodology proposed in this paper leads to the full use of available information and to potentially more accurate estimates for health impact assessment. In our case, lung cancer risks for diesel exhaust exposure were quantitatively remarkably similar for human studies and animal studies.

An early study used a Bayesian framework to combine dose response slopes from humans and animals from different species, different exposures and endpoints [3]. A more recent study used meta-analysis to combine human and animal data on the risks of chlorination byproducts [5]. Both studies did not adjust for biases in the individual studies [5].

Ordinary sensitivity analysis that was applied for the adjustment of confounding effects may be replaced by more advanced methods such as Monte Carlo risk analysis or Bayesian uncertainty assessment [16] which allow for the control of more than a single source of uncertainty at a time. However, no analysis captures every conceivable source of uncertainty. The goal of bias analysis is to adequately reflect the major sources through a range of possible estimates, while avoiding unimportant details.

The effect estimates of the animal and human studies were remarkably similar, suggesting that pooling the data may be useful. Rats have traditionally been used in many toxicological studies of the tumorigenic activity of biopersistent dusts. The US EPA has however decided against using the diesel exhaust lung cancer data obtained in rats for deriving a human cancer risk [21]. The main argument was that the development of lung tumors in rats is frequently attributed to ongoing inflammation and not to particle-specific toxicity, i.e. to the 'lung overload' phenomenon [36]. This phenomenon is an important mode of action (MOA) in rats at high concentrations, but such a mechanism is unlikely to be important in humans at low doses. The observation that no cancer risk was found in other species also contributed. Further uncertainties in using animal data include differences in exposure between laboratory and occupational settings, converting exposure duration and biological dose [37].

Even though the lung overload phenomenon is almost peculiar to the rat species, not every tumorigenic response should a priori be labelled as being a consequence of particle overload [38]. In particular, a critical review of the study data is needed before deciding whether the results of a chronic inhalation study fit the category of particle overload. The original studies data used for the purposes of the present study do not provide evidence of significant lung overload. Moreover, even if lung overload has indeed happened, previous studies have shown that overload seems also to occur in humans with heavy dust lung burdens, as for example coal workers (see e.g. [39]). Finally, extrapolation from animal to human has been based on all doses which provides greater confidence and is not limited to the highest doses where dose-response curves are poorly defined and lung tumor responses become saturated [36].

It is also important to note that combining evidence from human and animal studies into a common effect estimate should not be a standard practice. Consistency between the study-specific adjusted estimates is an important requirement for proceeding with meta-analysis. If such a condition is not satisfied, reporting the individual estimates or meta-analyzing separately the human and animal studies should be preferred.

Another limitation that concerns both bias adjustment and extrapolation from animal to human is the potential for the introduction of extra uncertainty through the (usually arbitrary) assumptions made for each scenario under consideration. These assumptions can significantly influence the value and quality of the bias-adjusted risk estimates and consequently the weights used to combine these adjusted estimates into a common effect measure. However such assumptions are inevitable and under reliable scenarios their effect could be negligible. For example, the use of appropriate time intervals, dose levels and disease groups or the choice of a reasonable dose-response curve can ensure that the extra uncertainty induced is minimal compared to other sources of bias in the original data and/or study design.

We should also note that the proposed methodology does not have a uniform application. In particular, special considerations are required depending on the environmental stressor and the outcome under study as well as the availability of data and the reliability of possible scenarios. For example, getting the relevant data on confounder prevalence and/or misclassification probabilities is not an easy task. In the first case, information on the confounder should also regard the time period of exposure and the population under study. Handling misclassification requires even more assumptions, the plausibility of which may be questionable in some cases. Moreover, using the original study's data in order to base such assumptions is frequently inevitable.

## Conclusions

In this work, we illustrate a methodology for combining evidence from bias-adjusted human and animal studies which is viable and provides a formal way of making efficient use of all the available information. This is particularly useful in health impact assessment since the effectiveness of intervention measures is strongly related to the strength and consistency of the available information. Especially in cases where evidence from epidemiological and toxicological studies is either contradictory or ambiguous, combining bias-adjusted effect estimates into a common measure could strengthen evidence for the characterization of pollutant-specific risks for human health.

## List of Abbreviations

AIC: Akaike Information Criterion; BMD: BenchMark Dose; BMDL: Benchmark Dose (Lower Confidence Limit); BMR: Benchmark Response; CB: Carbon Black; CI: Confidence Interval; DE: Diesel Exhaust; HCB: High Carbon Black; HDE: High Diesel Exhaust; I: Incidence; LCB: Low Carbon Black; LDE: Low Diesel Exhaust; LOAEL: Lowest Observed Adverse Effect Level; MCRA: Monte Carlo Risk Analysis; NO2: Nitrogen Dioxide; NOAEL: No Observed Adverse Effect Level; NOEL: No Observed Effect Level; OEL: Observed Effect Level; OR: Odds Ratio; PE: Exposure Prevalence; Pr: Prevalence; POD: Point Of Departure; RR: Risk Ratio; Se: Sensitivity; SMR: Standardized Mortality Ratio; Sp: Specificity

## Competing interests

The authors declare that they have no competing interests.

## Authors' contributions

XP studied existing DE and lung cancer related studies, selected the human and animal studies to be analyzed, performed the statistical analysis and drafted the main manuscript. GH and KK contributed expertise on risk assessment and methods to account for bias, participated in the design and coordination of the study and helped to draft the manuscript. All authors read and approved the final manuscript.

## References

[B1] U.S National Research Council Risk Assessment in the Federal Government: Managing the Process 1983 Washington, DC: National Academy Press 25032414

[B2] Calow P Handbook of Environmental Risk Assessment and Management 1998 Blackwell Science Ltd., Oxford, England

[B3] U.S National Research Council Science and Decisions: Advancing Risk Assessment 2009 Washington, DC: National Academies Press 25009905

[B4] DuMouchel WH Harris JE Bayes Methods for Combining the Results of Cancer Studies in Humans and Other Species Journal of the American Statistical Association 1983 78 293 308 10.2307/2288631

[B5] U.S National Research Council: Biological Effects of Ionizing Radiation (BEIR) IV report Health Effects of Radon and Other Internally Deposited Alpha Emitters 1988 National Academy Press, Washington, DC 25032289

[B6] Peters JL Rushton L Sutton AJ Jones DR Abrams KR Mugglestone MA Bayesian methods for the cross-design synthesis of epidemiological and toxicological evidence Journal of the Royal Statistical Society: Series C (Applied Statistics) 2005 54 159 172 10.1111/j.1467-9876.2005.00476.x

[B7] Kuempel ED Smith RJ Dankovic DA Stayner LT Rat- and human-based risk estimates of lung cancer from occupational exposure to poorly-soluble particles: A quantitative evaluation Journal of Physics: Conference Series 2009 151 012011 10.1088/1742-6596/151/1/012011

[B8] California Air Resources Board The Report on Diesel Exhaust. Findings of the Scientific Review Panel on the Report on Diesel Exhaust (As Adopted at the Panel's April 22, 1998 Meeting) 1998

[B9] U.S EPA Health assessment document for diesel engine exhaust 2002 National Center for Environmental Assessment - Office of Research and Development. Washington, DC EPA/600/8-90/057F Section 7.2

[B10] Mundt KA Tritschler JP II Dell LD Validity of Epidemiological Data in Risk Assessment Applications Human and Ecological Risk Assessment 1998 4 675 683 10.1080/10807039891284550

[B11] Health Effects Institute Diesel Exhaust: Critical Analysis of Emissions, Exposure and Health Effects 1995 Special Report

[B12] Green T Species Differences in Carcinogenicity: The Role of Metabolism in Human Risk Evaluation Teratogenesis, Carcinogenesis, and Mutagenesis 1990 10 103 113 10.1002/tcm.1770100206 1973849

[B13] Stayner L Dankovic D Smith R Steenland K Predicted Lung Cancer Risk Among Miners Exposed to Diesel Exhaust Particles American Journal of Industrial Medicine 1990 34 207 219 10.1002/(SICI)1097-0274(199809)34:3<207::AID-AJIM2>3.0.CO;2-S 9698989

[B14] Briggs DJ A framework for integrated environmental health impact assessment of systemic risks Environmental Health 2008 7 61 10.1186/1476-069X-7-61 19038020 PMC2621147

[B15] Knol AB Petersen AC van der Sluijs JP Lebret E Dealing with uncertainties in environmental burden of disease assessment Environmental Health 2009 8 21 10.1186/1476-069X-8-21 19400963 PMC2684742

[B16] Greenland S Multi-bias modelling for analysis of observational data Journal of the Royal Statistical Society A 2005 168 267 306 10.1111/j.1467-985X.2004.00349.x

[B17] Lipsett M Campleman S Occupational Exposure to Diesel Exhaust and Lung Cancer: A Meta-Analysis American Journal of Public Health 1999 89 1009 1017 10.2105/AJPH.89.7.1009 10394308 PMC1508841

[B18] Garshick E Laden F Hart JE Rosner B Smith TJ Dockery DW Speizer FE Lung Cancer in Railroad Workers Exposed to Diesel Exhaust Environmental Health Perspectives 2004 112 1539 1543 10.1289/ehp.7195 15531439 PMC1247618

[B19] Wellmann J Weiland SK Neiteler G Klein G Staif K Cancer mortality in German carbon black workers 1976-98 Occupational and Environmental Medicine 2007 63 513 521 10.1136/oem.2006.026526 PMC2078133 16497850

[B20] Gustavsson P Jakobsson R Nyberg F Pershagen G Järup L Schéele P Occupational Exposure and Lung Cancer Risk: A Population-based Case-Referent Study in Sweden American Journal of Epidemiology 2000 152 32 40 10.1093/aje/152.1.32 10901327

[B21] Ris C U.S. EPA Health Assessment for Diesel Engine Exhaust: A Review Inhalation Toxicology 2007 19 229 239 10.1080/08958370701497960 17886071

[B22] Olsson AC Gustavsson P Kromhout H Peters S Vermeulen R Brüske I Pesch B Siemiatycki J Pintos J Brüning T Cassidy A Wichmann HE Consonni D Landi MT Caporaso N Plato N Merletti F Mirabelli D Richiardi L Jöckel KH Ahrens W Pohlabeln H Lissowska J Szeszenia-Dabrowska N Zaridze D Stücker I Benhamou S Bencko V Foretova L Janout V Rudnai P Fabianova E Stanescu Dumitru R Gross IM Kendzia B Forastiere F Bueno-de-Mesquita B Brennan P Boffetta P Straif K Exposure to Diesel Motor Exhaust and Lung Cancer Risk in a Pooled Analysis from Case-Control Studies in Europe and Canada Am J Respir Crit Care Med 2010 21037020 10.1164/rccm.201006-0940OC PMC7259806

[B23] Steenland K Greenland S Monte Carlo Sensitivity Analysis and Bayesian Analysis of Smoking as an Unmeasured Confounder in a Study of Silica and Lung Cancer American Journal of Epidemiology 2004 160 384 392 10.1093/aje/kwh211 15286024

[B24] Jurek AM Greenland S Maldonado G Church TR Proper interpretation of non-differential misclassification effects: expectations vs. observations International Journal of Epidemiology 2005 34 680 687 10.1093/ije/dyi060 15802377

[B25] U.S. EPA Benchmark Dose Technical Guidance Document. [External Review Draft] 2000 EPA/630/R-00/001

[B26] Mauderly JL Relevance of Particle-induced Rat Lung Tumors for Assessing Lung Carcinogenic Hazard and Human Lung Cancer Risk Environmental Health Perspectives 1997 105 1337 1346 10.2307/3433557 9400748 PMC1470153

[B27] Laaksonen M Rahkonen O Karvonen S Lahelma E Socioeconomic status and smoking: Analysing inequalities with multiple indicators European Journal of Public Health 2005 15 262 269 10.1093/eurpub/cki115 15755781

[B28] Axelson O Steenland K Indirect methods of assessing the effects of tobacco use in occupational studies American Journal of Industrial Medicine 1988 13 105 118 10.1002/ajim.4700130107 3344750

[B29] Brightwell J Fouillet X Cassano-Zoppi AL Bernstein D Crawley F Duchosal F Gatz R Pfeifer H A framework for integrated environmental health impact assessment of systemic risks Journal of Applied Toxicology 1989 9 23 31 10.1002/jat.2550090106 2466883

[B30] Valberg PA Crouch EAC Meta-analysis of Rat Lung Tumors from Lifetime Inhalation of Diesel Exhaust Environmental Health Perspectives 1999 107 693 699 10.1289/ehp.99107693 10464067 PMC1566471

[B31] Nikula KJ Snipes MB Barr EB Griffith WC Henderson RF Mauderly JL Comparative Pulmonary Toxicities and Carcinogenicities of Chronically Inhaled Diesel Exhaust and Carbon Black in F344 Rats Fundamental and Applied Toxicology 1995 25 80 94 10.1006/faat.1995.1042 7541380 PMC7528960

[B32] Mauderly JL Jones RK Griffith WC Henderson RF McClellan RO Diesel Exhaust is a Pulmonary Carcinogen in Rats Exposed Chronically by Inhalation Fundamental and Applied Toxicology 1987 9 208 221 10.1016/0272-0590(87)90044-3 2443412

[B33] U.S Department of Health and Human Service The Surgeon General The Health Consequences of Smoking. Cancer and Chronic Lung Disease in the Workplace 1986 36 Table 11

[B34] Morfeld P Büchte SF McCunney RJ Piekarski C Lung Cancer Mortality and Carbon Black Exposure: Uncertainties of SMR Analyses in a Cohort Study at a German Carbon Black Production Plant Journal of Occupational and Environmental Medicine 2006 48 1253 1264 10.1097/01.jom.0000215344.77132.99 17159642

[B35] Büchte SF Morfeld P Wellmann J Bolm-Audorff U McCunney RJ Piekarski C Lung Cancer Mortality and Carbon Black Exposure: A Nested Case-Control Study at a German Carbon Black Production Plant Journal of Occupational and Environmental Medicine 2006 48 1242 1252 17159641 10.1097/01.jom.0000215710.17519.cd

[B36] Valberg PA Bruch J McCunney RJ Are rat results from intratracheal instillation of 19 granular dusts a reliable basis for predicting cancer risk? Regulatory Toxicology and Pharmacology 2009 54 72 83 10.1016/j.yrtph.2009.02.008 19275925

[B37] Valberg PA Watson AY Analysis of Diesel-Exhaust Unit-Risk Estimates Derived from Animal Bioassays Regulatory Toxicology and Pharmacology 1996 24 30 44 10.1006/rtph.1996.0062 8921544

[B38] Oberdörster G Lung Particle Overload: Implications for Occupational Exposures to Particles Regulatory Toxicology and Pharmacology 1994 27 123 135 10.1006/rtph.1995.1017 7784625

[B39] Morrow PE Possible mechanisms to explain dust overloading of the lungs Fundam Appl Toxicol 1988 10 369 384 10.1016/0272-0590(88)90284-9 3286345

